# Mathematical algorithm for the automatic recognition of intestinal parasites

**DOI:** 10.1371/journal.pone.0175646

**Published:** 2017-04-14

**Authors:** Alicia Alva, Carla Cangalaya, Miguel Quiliano, Casey Krebs, Robert H. Gilman, Patricia Sheen, Mirko Zimic

**Affiliations:** 1 Unidad de Bioinformática, Laboratorios de Investigación y Desarrollo, Facultad de Ciencias y Filosofía, Universidad Peruana Cayetano Heredia, Lima, Perú; 2 Laboratorio de Inmunopatología en Neurocisticercosis, Laboratorio de Investigación y Desarrollo, Facultad de Ciencias y Filosofía, Universidad Peruana Cayetano Heredia, Lima, Perú; 3 Facultad de Medicina Humana, Universidad Nacional Mayor de San Marcos, Lima, Perú; 4 Weill Cornell Medical College in New York City, New York, United States of America; 5 Department of International Health, School of Public Health, Johns Hopkins University, Baltimore, Maryland, United States of America; Seoul National University College of Medicine, REPUBLIC OF KOREA

## Abstract

Parasitic infections are generally diagnosed by professionals trained to recognize the morphological characteristics of the eggs in microscopic images of fecal smears. However, this laboratory diagnosis requires medical specialists which are lacking in many of the areas where these infections are most prevalent. In response to this public health issue, we developed a software based on pattern recognition analysis from microscopi digital images of fecal smears, capable of automatically recognizing and diagnosing common human intestinal parasites. To this end, we selected 229, 124, 217, and 229 objects from microscopic images of fecal smears positive for *Taenia* sp., *Trichuris trichiura*, *Diphyllobothrium latum*, and *Fasciola hepatica*, respectively. Representative photographs were selected by a parasitologist. We then implemented our algorithm in the open source program SCILAB. The algorithm processes the image by first converting to gray-scale, then applies a fourteen step filtering process, and produces a skeletonized and tri-colored image. The features extracted fall into two general categories: geometric characteristics and brightness descriptions. Individual characteristics were quantified and evaluated with a logistic regression to model their ability to correctly identify each parasite separately. Subsequently, all algorithms were evaluated for false positive cross reactivity with the other parasites studied, excepting *Taenia* sp. which shares very few morphological characteristics with the others. The principal result showed that our algorithm reached sensitivities between 99.10%-100% and specificities between 98.13%- 98.38% to detect each parasite separately. We did not find any cross-positivity in the algorithms for the three parasites evaluated. In conclusion, the results demonstrated the capacity of our computer algorithm to automatically recognize and diagnose *Taenia* sp., *Trichuris trichiura*, *Diphyllobothrium latum*, and *Fasciola hepatica* with a high sensitivity and specificity.

## Introduction

Intestinal parasites are among the most common infectious diseases in humans worldwide, with a higher prevalence in developing countries and economically depressed communities. As such, these infections are considered to be a product of poor living conditions the impact of which is frequently underestimated by public health services. Nevertheless, in the last few years the role of these infectious agents, especially on the long term physical and mental development of children, has been increasingly recognized [1, 2]. This recognition presents the challenge to search for a sustainable and cost-effective solution to this problem. In order for public health authorities to monitor the epidemiologic distribution and variation of the parasites, and to develop appropriate control methods, an effective diagnostic tool is needed to correctly identify the parasites and determine prevalence and incidence [2, 3].

Previous studies on pattern recognition in images in the field of parasitology were developed to help diagnose medically relevant parasites [4, 5]. The methodology used can be divided into three general categories: pre-processing, image processing with feature extraction, and classification [6, 7].

In order to increase the capacity of a computer algorithm to account for nuanced differences between parasite eggs, increasingly complex systems have been used. Previous studies have reported the use of artificial neural networks (ANN) [8], adaptive network based fuzzy inference system (ANFIS) [9], Multi-Class Support Vector Machine (MCSVM) classifier [10], Bayesian classification system [11] and in order to identify and classify parasites. More recently, a technique to detect parasites in fecal smears used the Filtration and Steady Determinations Thresholds System (F-SDTS) to segment and classify two different parasitic eggs, Platyhelminthes and Nematodes, with an overall correct classification rates of 93% and 94%, respectively [12].

All of these works recognize the helminth egg by extracting their morphologic characteristics from images; the same characteristics used by specialists to diagnose parasites in fecal smears. The actual diagnosis from a stool sample requires expert personnel, which may overburden specialists in areas of high parasitemia. For this reason, emerging automated computer algorithms could play an important role in supporting the clinical diagnosis of intestinal parasitemia.

The present work discusses our development of a software to automatically diagnose *Taenia* sp., *Fasciola hepatica*, *Diphyllobothrium latum* and *Trichuris trichiura*—prevalent human parasites—from microscopic digital images of stool samples. We developed a computer algorithm to process the images, extract features and subsequently classify images through a simple multivariate logistic regression based on the most representative features of the eggs of each parasite.

## Materials and methods

### Sample selection

The study which provides the stool samples was performed at the Universidad Peruana Cayetano Heredia, Lima, Peru, under the protocol “Evaluación de infecciones gastroinstestinales por norovirus y parasitos en comunidades urbano marginales del Peru”, with Dr. Robert Gilman as principal investigator. This study was approved by the IRB Human Ethics of the Universidad Peruana Cayetano Heredia -CEIH (Constancia de autorización ética No. CP52240, February 2007) which complies with the Office for Human Research Protections—OHRP (Federalwide Assurance: FWA00000525). The samples used in this study were stored at 4°C in the Parasitology Laboratory at Universidad Peruana Cayetano Heredia, were preserved in phosphate-buffered saline–formaldehyde at 5% and de-identified prior to the parasitological examination.

We used 200 stool samples which were processed using rapid sedimentation (centrifugation) to concentrate any parasitic eggs present in the samples [13]. Following this sedimentation step, samples were stained with Lugol’s iodine and examined under the microscope. A laboratory technician prepared the samples and slides, and a parasitology specialist made the diagnoses and took photographs of each slide [13].

Each photo selected for this study contains only one parasite egg. The dataset with the positive identifications by specialists consisted of 349 *Taenia* sp., 124 *Trichuris trichiura*, 185 *Diphyllobothrium latum*, and 460 *Fasciola hepatica* photos/egg. Additionally, we selected 462 image artifacts with negative identifications.

### Image digitalization

We used an optic microscope (DIALUX^®^ Leitz Weitz) outfitted with a 3.34 megapixel camera (Olympus C-3030) to digitalize the fecal smears at 40x magnification. For all photos, we used the light source at maximum intensity with a diffusor and collimator to prevent punctate light artifacts, and captured the images with the camera at its maximum optical zoom (3.34 megapixels), with autofocus but without flash.

### Image processing

**Fig 1:**
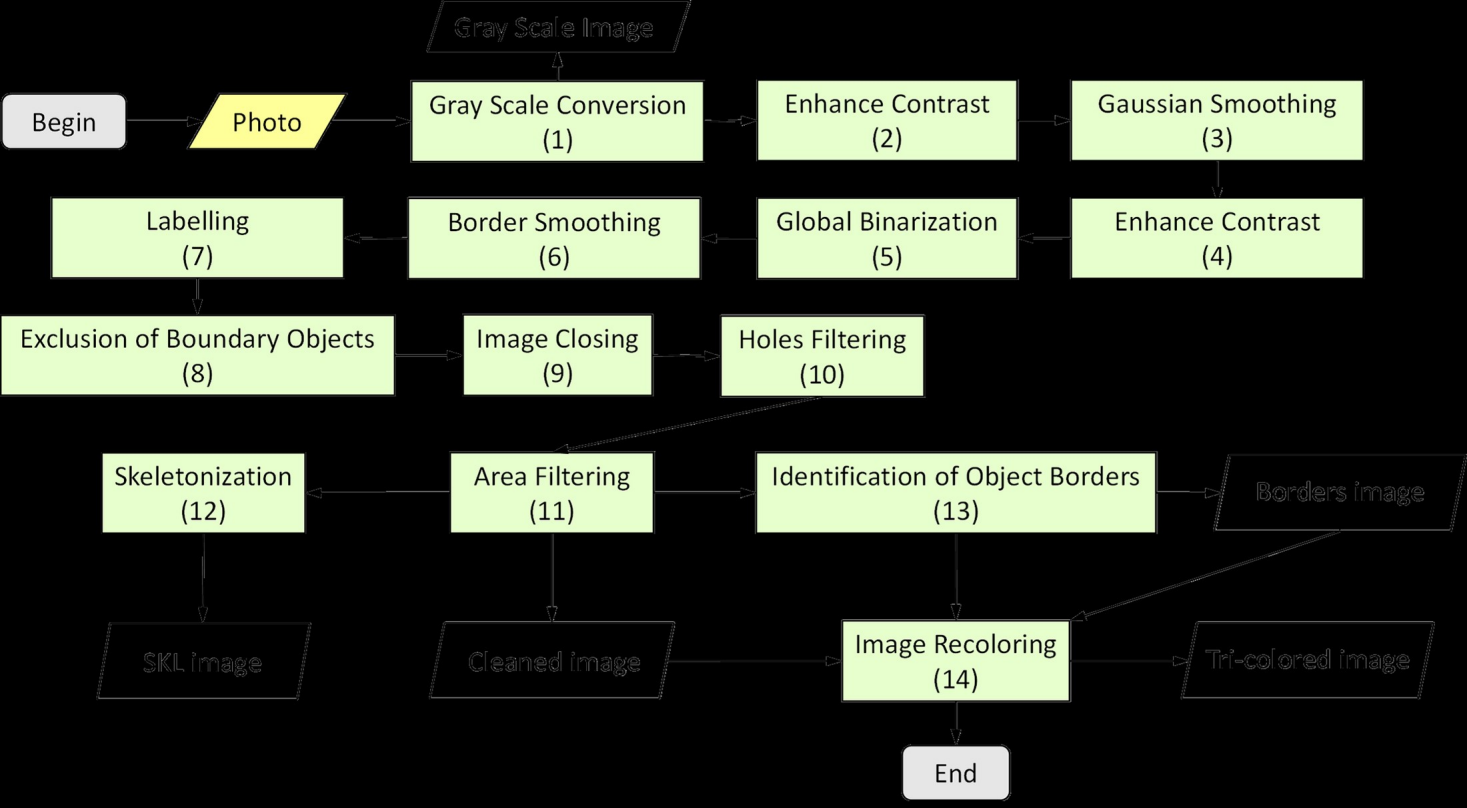
The processing flow of the image processing for parasite eggs. The initial input is the original image of the eggs, captured at 40x magnification. Fourteen steps enhance contrast and filter out noise in order to obtain the final images that serve as inputs for the feature extraction process.

**Fig 2:**
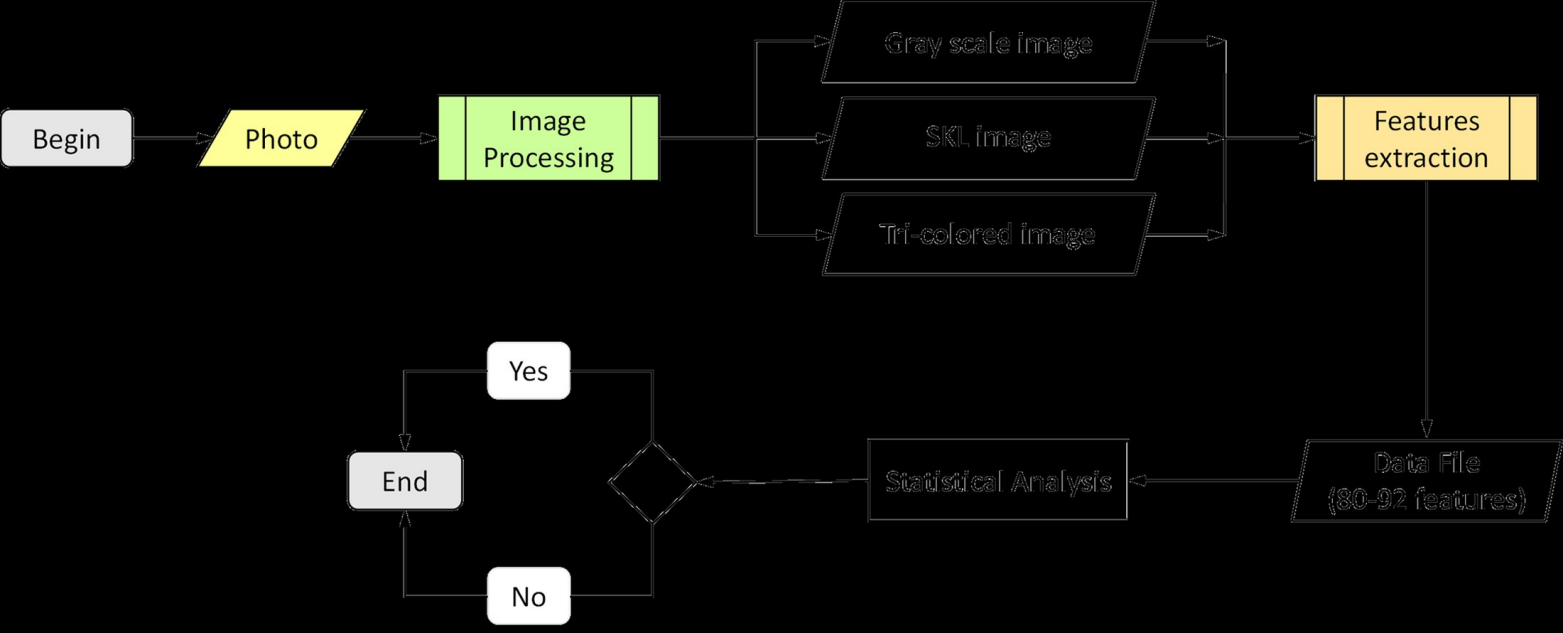
Flowchart of proposed methodology to automatically recognize parasitic eggs from microscopic photographs.

We developed our algorithm in SCILAB, an open source computational platform, to process and extract features from our images. The sequential steps, previously described in our publication on automatic recognition of *Mycobacterium tuberculosis* cords, were used to process all images [14]. Two additional steps were added in order to increase the contrast (enhance contrast) and reduce background noise (Gaussian smoothing). These steps consisted of the successive application of different filters and masks. We applied the following fourteen steps to process each digital image: gray scale conversion, enhance contrast, Gaussian smoothing, enhance contrast, global binarization, border smoothing, labelling, exclusion of boundary objects, image closing, holes filtering, area filtering, skeletonization, identification of object borders, and image recoloring (Fig 1). Five different images were produced during the processing of each original photograph: gray-scale, skeleton image, border image, cleaned image, and a tri-color image—this final image being constructed by combining the cleaned and border images (S1–S4 Figs). As such, the tri-color image was used in the feature extraction steps in place of the cleaned and border images (Fig 2). The skeleton image was composed of a trunk and branches (S1g–S4g Figs).

### Feature extraction and pattern recognition

The feature extraction steps considered different morphologic characteristics: geometric descriptions, curvature, internal patterns, and brightness-distribution using the skeleton, gray scale, and tri-colored images [15]. The elemental shared features of all parasites were the length, thickness, brightness distribution, circularity, and shape of the object [16].

#### Length of the object

Using tri-colored and skeleton images we can identify the long axis of each object and measure the branches extended to the edge of the object border. The length of the object was the total number of pixels that form the trunk extended to each edge.

#### Thickness of the object

Using the skeleton and tri-colored images, (border-skeleton correspondence) we drew transverse lines perpendicular to the trunk of the object from the skeleton image (transversal segmentation) at regular intervals. The thickness was expressed as the mean length of all transverse lines.

#### Brightness distribution of the object

Brightness measures were obtained using the gray scale image. The spatial variation of the brightness in the object was measured within each segment created by the perpendicular transverse lines. The increase in percent brightness, as compared to the mean brightness of the photograph, was analyzed using a series of cutoff values: 8%, 10%, 12%, 15%, 18%, 20% and 22%.

#### Circularity of the object

We measured circularity using the skeleton and the tri-colored images (border-skeleton correspondence). The circularity parameter was calculated for each object as 4π times its area divided by the square of its perimeter. The perimeter was calculated as the length of the border of the object.

#### Shape of the object

Using the skeleton and tri-colored images, we identified discrete points of the extended trunk that fit into a continuous non-linear function using a Gaussian fit. Then the curvature value of each point was estimated by the Fourier’s Fast Transformation (FFT). Next we identified waves, which we defined as any segment of the extended trunk that fell between a point with a curvature equal to or less than 0.2 and a second point with a change of curvature more than 0.4. Finally we estimated three variables: number of waves, size, and curvature of each wave (the maximum value of curvature in the wave).

For each parasite, we quantified additional features in order to encompass all of its most distinguishing characteristics.

### Mathematical variables for *Taenia* sp.

**Fig 3:**
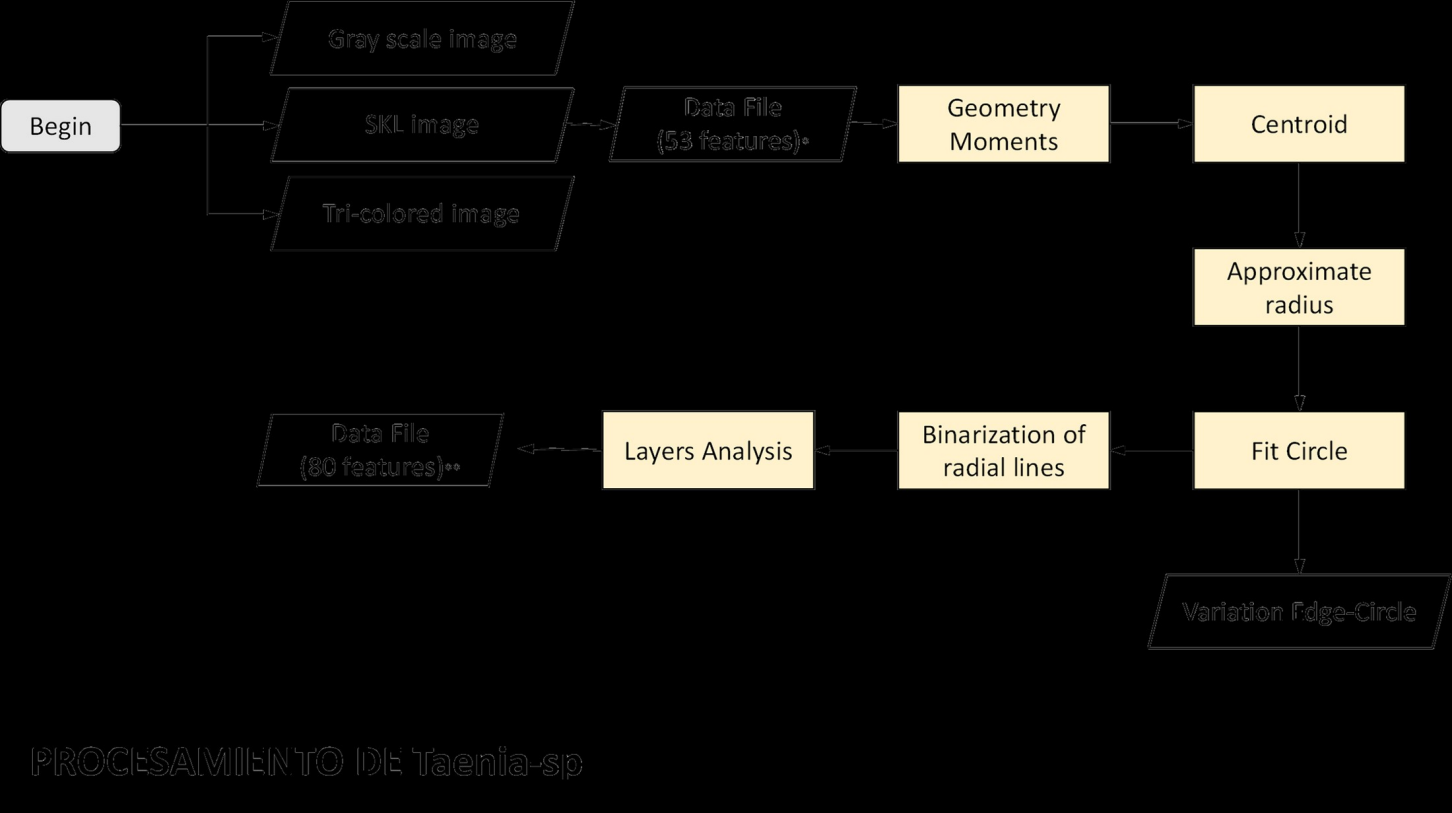
Flowchart of the feature extraction for *Taenia* sp. Flowchart showing feature extraction for *Taenia* sp. eggs, resulting in 80 variables for statistical analysis.

The eggs of *Taenia* sp. are spherical and measure 35–43 μm in diameter. Under light microscopy, the ova appear light brown, with a thick shell and characteristic radial striations, and contain a central hexagonal oncosphere with three pairs of hooklets [17]. Our algorithm analyzing the *Taenia* sp. egg centers on these morphological characteristics and the presence of radial layers. To obtain this additional variable for the layers, our algorithm first calculates geometric moments, the centroid of the object, and the average radius (mean length of uniformly spaced radial lines drawn from the calculated center of the object). The program also draws a best-fit circle around the *Taenia* sp. egg, and then calculates the difference between the original edge of the object and the computer-drawn best-fit circle in order to quantify the circularity of the egg with the ‘variation edge-circle’ variable. Additionally, for the *Taenia* sp. parasite egg we introduced a layers analysis by binarizing radial segments using Otsu thresholding. With uniformly distributed radial lines, we compared the most external portion of the resulting slices (outer 60% of each slice) and used the Otsu threshold to compare the pattern of light and dark layers of the outer shell, thus mathematically representing the radial striations characteristic of this shell. This entire analysis of *Taenia* sp. ova resulted in a data set consisting of 80 features (Fig 3).

### Mathematical variables for *Fasciola hepatica*

**Fig 4:**
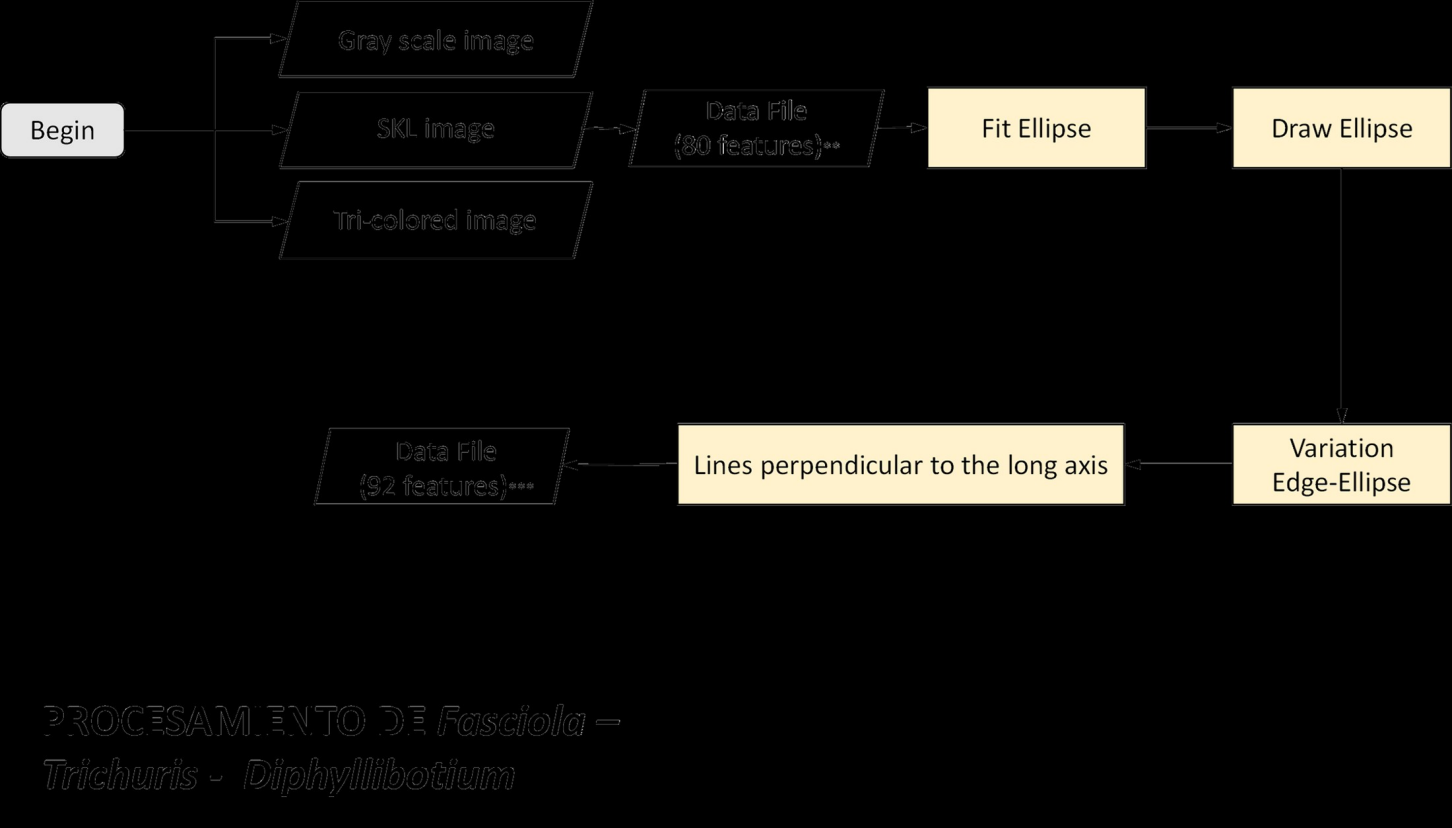
Flowchart of the feature extraction process for *Fasciola hepatica*, *Diphyllobothrium latum*, and *Trichuris trichiura* eggs. This process results in 92 features for statistical analysis.

The eggs of *Fasciola hepatica* are spheroids with diameters that usually measure between 130–150 μm by 63–90 μm and an operculum at one pole. The analysis of the morphological characteristics of the parasite *Fasciola hepatica* centers on the shape and internal texture of the egg [17]. On the digital images, our algorithm first drew a best-fit ellipse around the border of the egg. To numerically represent the shape, we measured the eccentricity of the best-fit ellipse, eccentricity in terms of moments, circumference of the best-fit ellipse, the difference between this drawn ellipse and the original border of the object, and the standard deviation between the two edge measurements (Fig 4). The internal structure of the parasite was analyzed by first identifying the major axis of the best-fit ellipse and then dividing the ellipse into radial sections with a uniform distribution from the major axis of the best-fit ellipse to the edge of the original image. With this grid, we analyzed the intensity of illumination and variations in light along the long axis of the parasite. Finally, as *Fasciola hepatica* is one of the largest parasites, we calculated the overall area of the object.

### Mathematical variables for *Diphyllobothrium latum*

The operculated, spheroid egg of this cestode usually measures between 58–75 μm by 45–50 μm. The ova are not embryonated in the feces, and the operculum forms a layer on one end which opens to expel an oncosphere [17]. The analysis of the morphological characteristics of the *Diphyllobothrium latum* is also based on the form and internal texture of the egg, and as such uses the same variables as described above for *Fasciola hepatica*. Again, we considered the overall area, as the size is the major differentiating variable between this parasite and *Fasciola hepatica* (Fig 4).

### Mathematical variables for *Trichuris trichiura*

The typical egg of *Trichuris trichiura* measures between 50–55 μm by 22–24 μm, is barrel-shaped with a characteristic plug at both ends, and contains a single monocellular ovum [17]. The analysis of the morphological features of the parasite *Trichuris trichiura* considers the shape, internal texture, and the area of the egg, which are the same variables as those used for *Fasciola hepatica* (Fig 4). *Trichuris trichiura* does possess an additional differentiating characteristic—transverse layers near the center of the parasite—thus we used an additional variable to analyze these layers. Using a sample space of transverse lines drawn along the major axis of the best-fit ellipse, we calculated this new variable. To take the protruding bipolar end plugs into account, we used the deviation of the border along the major axis of the best-fit ellipse from the actual border of the object.

### Statistical analysis

We elected to use a multiple logistic regression to analyze the potentially predicting variables for the four parasites, and selected the individual variables using a forward stepwise procedure, based on the initial univariate analysis. We evaluated the models using the R-squared value for degree of fit, odds ratio, capacity of the regression to make a correct classification (sensitivity, specificity, and area under the curve), and with maximum parsimony. Multicollinearity between the predictive variables was assessed with a Pearson correlation coefficient; if the absolute value of the Pearson correlation coefficient is greater than 0.75, then collinearity is very likely to exist and indicate that variables may be redundant. Such variables were excluded from the full multivariate logistic model. We evaluated the exclusivity of the regressions for each parasite by measuring the capacity of the model to detect the egg of the specific parasite while excluding the other parasites. For cross-reaction, each parasite model (*Trichuris trichiura*, *Diphyllobothrium latum*, and *Fasciola hepatica)* was tested in the object databases from the other parasites. All the statistical analyses were performed with a 5% significance level.

## Results

### *Taenia* sp.

**Fig 5:**
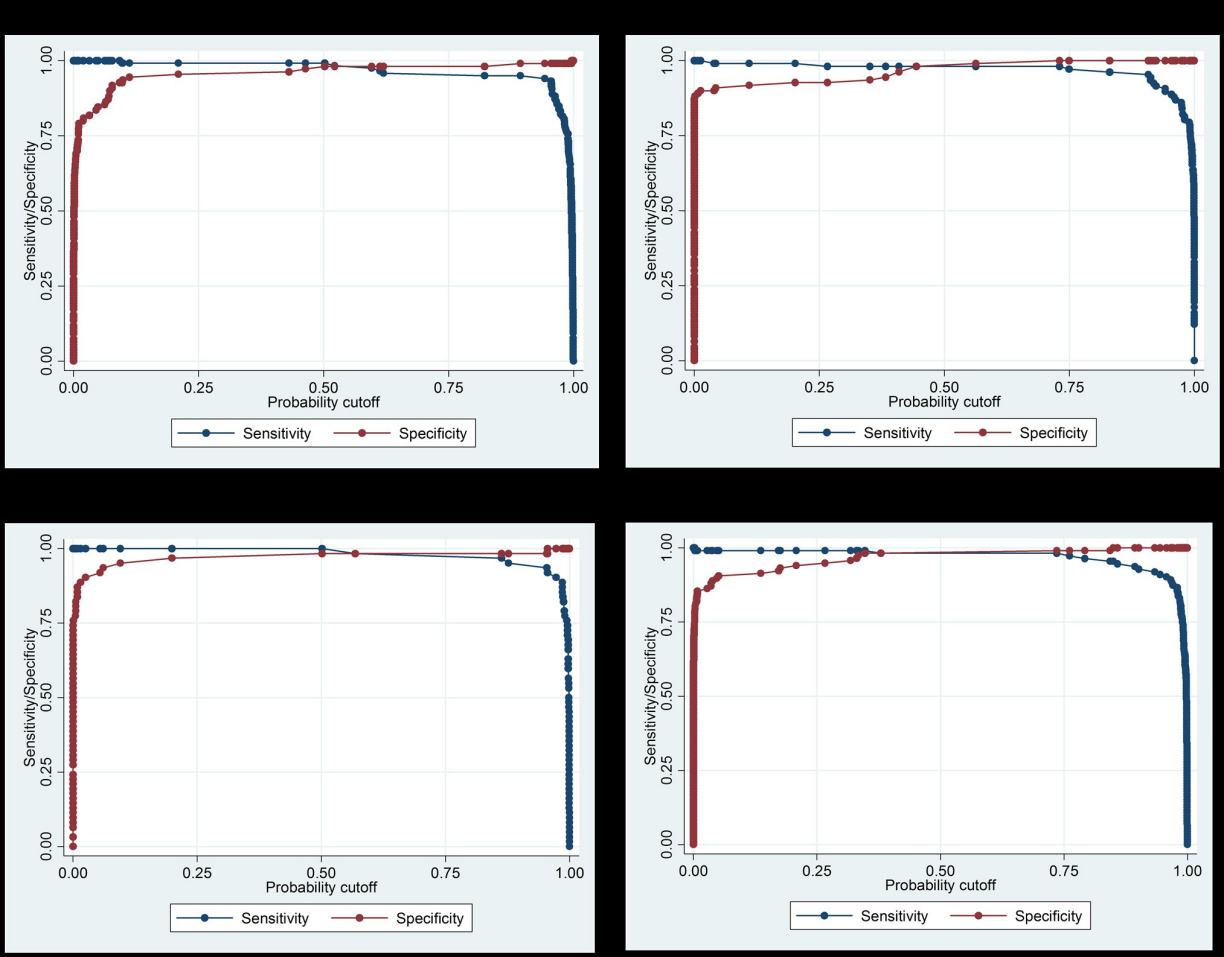
Sensitivity and specificity of each regression model’s ability to recognize parasites in digital images of fecal smears given differing probability cutoff values.

The most relevant variables were the circularity (geometric measure), and three quantifiers of the internal structure Optimal_threshold_ds, and tam_work (Table 1). We modeled the probability of correct identification of a *Taenia* sp. eggs in a multivariate logistic regression, obtaining a sensitivity and specificity of 99.10% and 98.29%, respectively. The proportion of total variance explained by this model was 91.3%. To further evaluate the overall precision of the model (Table 2), we calculated Youden’s index (0.974) and the area under the curve (0.9975) (Fig 5).

**Table 1:** Summary of the principle variables and the features they represent.

Dimension	Variable	Description
**Geometric descriptions**		
	*Circularity	Circularity of the digital object, values range from 0–1 and with values closer to 1 being a perfect circle.
	Minor axis	Length of the minor axis (shortest diameter) on the best fit ellipse.
	Major axis	Length of the major axis (longest diameter) on the best fit ellipse.
	Major/Minor axis	Ratio between the major and minor axes in the best fit ellipse.
	Diff perimeter	Difference between the perimeters of the best fit ellipse and the original object.
	Mean edgediffercircle	The average of the edge difference between the original object perimeter and the best-fit circle. This calculation is based on two images: the original object and best-fit circle. Using these images we traced uniformly distributed lines with the same orientation from the center of each object to different points on the edge. Next, we found the absolute value of the differences between each line and calculated the average difference.
	SD edgediffercircle	The standard deviation of the edge difference between the original object border and the best-fit circle. Using the same methodology for the variable Mean edgediffercircle, we calculated the standard deviation of the absolute value of all the differences between the original object and the best-fit circle.
	SD edgedifferellipse	The standard deviation of the absolute value of the difference between the edge of the original object and the best fit ellipse. We use the same methodology for the variable ds_difercirbor, except using an ellipse instead of circle.
**Quantification of the internal structure**		
	tam_work	The average of the length of the segments of the radial lines drawn to analyze the layers. This calculation was based on the lines drawn from the center of the original object to the edge of that object, and we used only the outer 60% of each line to identify the shell and area of radial striations. We also calculated the average length of each segment.
	tam_inici_ds	The standard deviation of the length of the radial lines drawn to analyze the layers. Using the same lines drawn for the calculation of the tam_work variable, we calculated the standard deviation of the lengths.
	Optimal_threshold_ds	The standard deviation of the Otsu threshold found in each segment of the radial lines drawn to analyze layers. We use the same segments as in the methodology for the calculation of the tam_work variable.
	Thickness	The average of the lengths of the perpendicular lines drawn from the skeleton image backbone to the border of the object.
	Thickness/length	The ratio between the thickness and the length of the object.
	Longest/total length	The ratio of the longest perpendicular line drawn transverse to the skeleton image backbone and the length of the object.
**Characterization of the curvature**		
	Curvature	The average of the curvature of the total waves in the object.
	Shape	The number of waves multiplied by the square of the curvature.
	Shape2	Square of shape.
**Brightness-distribution**		
	*Mean-Nrefr15	Average of the number of pixels along the transverse segment to the skeleton that has brightness 15% higher than the average brightness of the photograph (when normalized by the total number of pixels of that particular transverse segment).

* Variables have been previously reported in our publication on automatic recognition of *M*. *tuberculosis* in MODS [14].

**Table 2:** Summary of classification variables, regression model fit, and odds ratio of each regression’s ability to make an accurate classification.

Parasite Model	Features	Univariate logistic regression			Multivariate logistic regression		
		R^2^	Odds Ratio	p-Value	Overall R^2^	Odds Ratio	p-Value
*Taenia* sp.							
	Circularity	0.77	169227.3	<0.001	0.913	1244.9	0.003
	umbral_optimo_ds	0.15	3.50E-12	<0.001		5.01E-34	0.015
	Mean edgediffercircle	0.66	0.73	<0.001		0.82	0.011
	tam_work	0.55	1.21	<0.001		1.1	0.038
*Fasciola hepatica*							
	SD edgediffercircle	0.73	1.18	<0.001	0.898	1.08	0.006
	Diff perimeter	0.15	0.95	<0.001		0.91	0.054
	Major/Minor axis	0.08	2.22	<0.001		9.36	0.017
	Curvature	0.4	0	<0.001		0	0.005
	Longest/total length	0.32	22641.76	<0.001		46831.97	0.01
*Diphyllobothrium latum*							
	Thickness/length	0.6	1.14	<0.001	0.92	1.13	0.008
	Minor axis	0.73	1.1	<0.001		1.52	0.002
	tam_inici_ds	<0.001	1	<0.874		0.61	0.003
	Mean-Nrefr15	0.22	1.13	<0.001		1.11	0.04
	Shape	0.004	1	0.293		1.04	0.027
*Trichuris trichura*							
	Shape2	0.57	0.94	<0.001	0.937	0.88	0.024
	Major axis	0.24	1.02	<0.001		1.15	0.006
	SD edgedifferellipse	0.14	0.91	<0.001		0.35	0.006
	Thickness	0.45	1.05	<0.001		0.84	0.008

### Fasciola hepatica

The most relevant variables were the SD edgediffercircle, Diff perimeter, Major/Minor axis, Curvature, and Longest/total length (Table 1). The pseudo R-squared value for our best logistic model was 0.898 (Table 2), with a sensitivity of 99.15% and specificity of 98.18%. The precision (Youden’s index) was 0.9734 and area under the curve was 0.9948 (Fig 5).

### Diphyllobothrium latum

The most relevant variables were the Thickness/length, Minor axis, tam_inici_ds, Mean-Nrefr15, and Shape (Table 1). The best logistic regression model had 100% and 98.13% sensitivity and specificity, respectively. The proportion of the total variance explained by the model was 92% (Table 2), precision (Youden’s index) was 0.9813, and the area under the curve was 0.9984 (Fig 5).

### Trichuris trichura

Shape2, major axis, SD edgedifferellipse, and thickness (Table 1). The best logistic regression model obtained a sensitivity of 100%, and specificity of 98.38%. The proportion of variance in the outcome explained by this model was 93.7% (Table 2), Youden’s index was 0.9838, and the area under the curve was 0.9987 (Fig 5).

The models showed no cross-reactions among the other parasites studied.

## Discussion

The diagnosis of intestinal parasites in actuality is a qualitative process, based on the microscopic identification of the form of the parasite in fecal samples, and depends on the experience of the observer. Generally, parasites are identified by specialized health professional; nevertheless, not all health clinics in the country have access to those specialists. Given the need to facilitate diagnosis, we propose an algorithm to automatically recognize intestinal parasites without physician input.

Automatic segmentation during image processing of the eggs is crucial for the feature extraction and classification stage. Many studies have reported the automated segmentation from the image background step as being very inconvenient, and had to rely on manual segmentation [5], on automatic segmentation using threshold operations [8, 9, 11, 18] or with successive steps combining different filters [15, 19]. In this study, the images of the parasites were captured with a high background, directly from fecal samples without any preprocessing. To resolve the significant noise interference and improve the segment performance we propose a new image processing methodology. This methodology has been previously used and published for the automatic detection of tuberculosis in sputum samples [14]. The principle differences are basically two filters that enhance the contrast and remove background noise using Gaussian smoothing, which are summarized in the 12 successive filters described in our methods. They are able to successfully reduce background noise and achieve an improved segmentation of each object, thus better defining the parasitic ova in the images. This automatic segmentation of the objects of interest corresponds with an improvement in the quality of the morphological characteristics extracted, leading to a better statistical analysis and classification.

Automatic recognition of the egg of intestinal parasites has been investigated in different studies based on the geometric shape, curvature, and texture of the egg. Many of these other studies have employed different classification methodologies using multivariate statistics [4, 5], Bayesian classification [11], artificial neural networks (ANN) [8], multi-class support vector machines (MCSVM) [10], a fuzzy inference system based on adaptive network (ANFIS) [9], filtration with steady determinations thresholds System (F-SDTS) [12], and multi-texton histogram (MTH) [20]. The 4 algorithms presented in this study also consider many of the same morphological characteristics, but using a simpler logistic regression model in order to classify and model the probability of a positive *Taenia* sp., *Fasciola hepatica*, *Diphyllobothrium latum*, and *Trichuris trichuria* egg. Our system using logistic regression as classification methodology identified parasitic eggs with a high sensitivity and specificity.

Previous studies on automatic recognition of parasites describe methodologies that demonstrate their ability to recognize different parasites, yet do not report the occurrence of any cross-reactivity in the algorithms for other parasite types. In our study, we evaluated cross-reactivity between *Fasciola hepatica*, *Diphyllobothrium latum*, and *Trichuris trichuria*, which revealed no false positive identifications. *Taenia* sp. was not evaluated for cross-reactivity as it does not share the same extracted features with the other three parasites.

The results obtained in the present study show that it is possible to diagnose certain intestinal parasites using microscopic digital images with a high accuracy. The sensitivity and specificity were 99.10% and 98.29%, 99.15 and 98.18%, 100% and 98.13%, and 100% and 98.38% for *Taenia* sp., *Fasciola hepatica*, *Trichuris trichuria*, and *Diphylobotrium latum* respectively. As presented here, our 4 algorithms function in the open-source computational program Scilab is capable of automatically recognizing human intestinal parasites. Given the widespread nature of these parasitic infections in areas with low resources, we hope to contribute to automated methods of surveying, diagnosing, and treating this disease.

## Supporting information

S1 Fig Steps of image processing of *Taenia* sp. Process: (a) Original image, (b) gray-scale image, (c) image with contrast filter, (d) binarized image, (e) object filling and border smoothing, (f) area filter and background coloration, (g) digital object skeleton and (h) drawn border.
(TIF)

S2 Fig Steps of image processing of *Fasciola hepatica*. Process: (a) Original image, (b) gray-scale image, (c) image with contrast filter, (d) binarized image, (e) object filling and border smoothing, (f) area filter and background coloration, (g) digital object skeleton and (h) drawn border.
(TIF)

S3 Fig Steps of image processing of *Diphyllobothrium latum*. Process: (a) Original image, (b) gray-scale image, (c) image with contrast filter, (d) binarized image, (e) object filling and border smoothing, (f) area filter and background coloration, (g) digital object skeleton and (h) drawn border.
(TIF)

S4 Fig Steps of image processing of *Trichuris trichiura*. Process: (a) Original image, (b) gray-scale image, (c) image with contrast filter, (d) binarized image, (e) object filling and border smoothing, (f) area filter and background coloration, (g) digital object skeleton and (h) drawn border.
(TIF)
