# Supplementary figures and images for: Mathematical algorithm for the automatic recognition of intestinal parasites
Source: PLoS One. 2017 Apr 14;12(4):e0175646. doi: 10.1371/journal.pone.0175646 (PMC5391948; doi:10.1371/journal.pone.0175646)

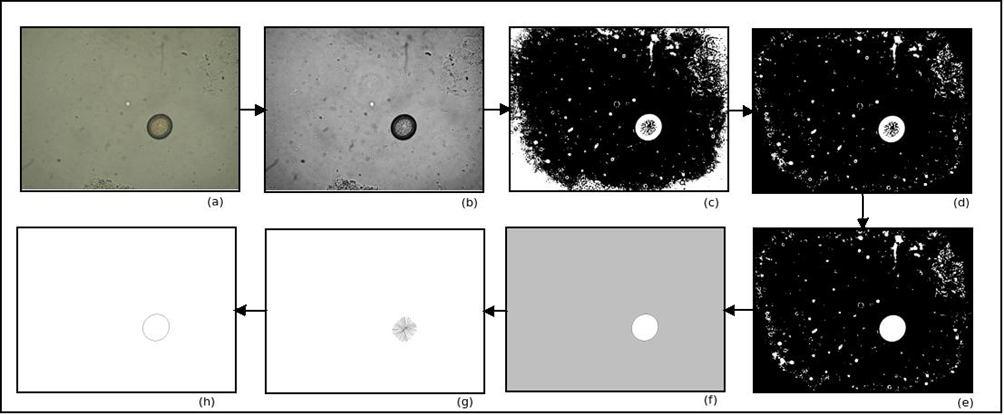

Supplement: S1 Fig — Process: (a) Original image, (b) gray-scale image, (c) image with contrast filter, (d) binarized image, (e) object filling and border smoothing, (f) area filter and background coloration, (g) digital object skeleton and (h) drawn border. (TIF) [file pone.0175646.s001.tif]

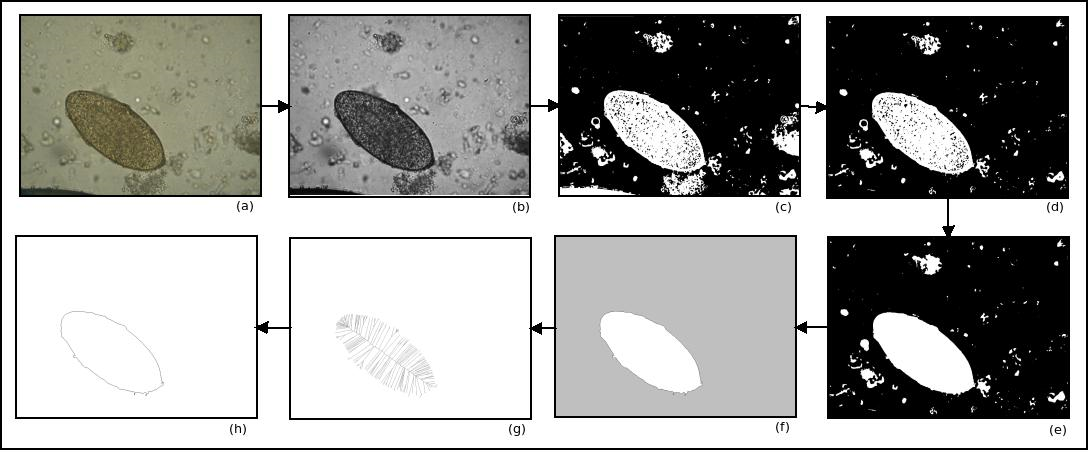

Supplement: S2 Fig — Process: (a) Original image, (b) gray-scale image, (c) image with contrast filter, (d) binarized image, (e) object filling and border smoothing, (f) area filter and background coloration, (g) digital object skeleton and (h) drawn border. (TIF) [file pone.0175646.s002.tif]

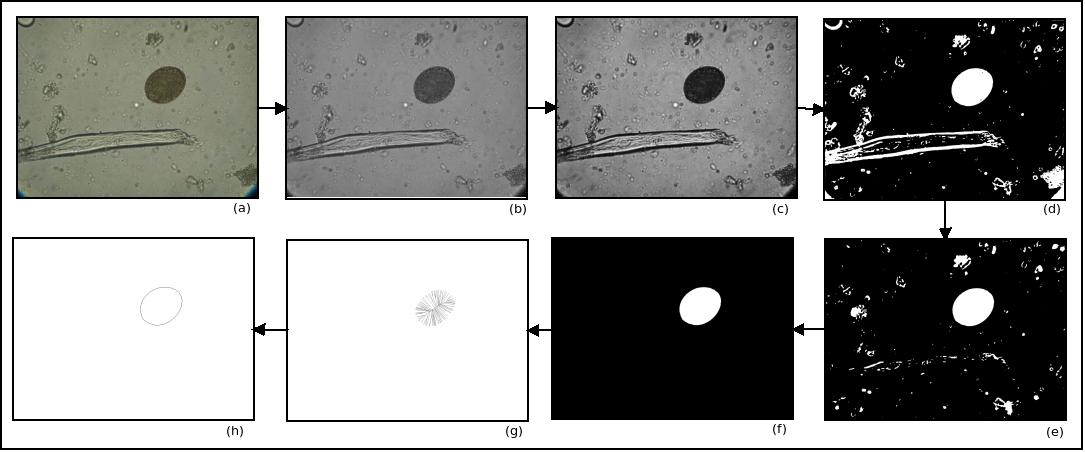

Supplement: S3 Fig — Process: (a) Original image, (b) gray-scale image, (c) image with contrast filter, (d) binarized image, (e) object filling and border smoothing, (f) area filter and background coloration, (g) digital object skeleton and (h) drawn border. (TIF) [file pone.0175646.s003.tif]

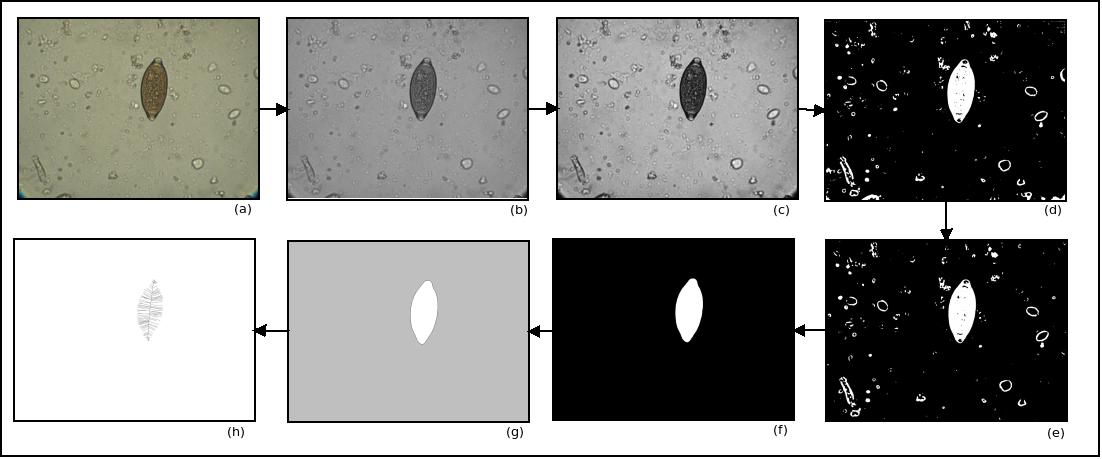

Supplement: S4 Fig — Process: (a) Original image, (b) gray-scale image, (c) image with contrast filter, (d) binarized image, (e) object filling and border smoothing, (f) area filter and background coloration, (g) digital object skeleton and (h) drawn border. (TIF) [file pone.0175646.s004.tif]
